# Supplementary material for: Brazilian women’s use of evidence-based practices in childbirth after participating in the Senses of Birth intervention: A mixed-methods study
Source: PLoS One. 2021 Apr 16;16(4):e0248740. doi: 10.1371/journal.pone.0248740 (PMC8051805; doi:10.1371/journal.pone.0248740)
Supplement: S1 File — (DOCX) [file pone.0248740.s001.docx]

# **S1 File – The Senses of Birth Intervention and the Theory of Planned Behavior**

Figure 1 – Proposed theoretical framework of Senses of Birth Intervention and its impact on birth outcome [1]

[1] L. da M. M. Fernandes, “Pregnant women’s knowledge and use of evidence-based practices during labor and childbirth after participating in a health education intervention – Senses of Birth,” State University of New York, 2019.

**
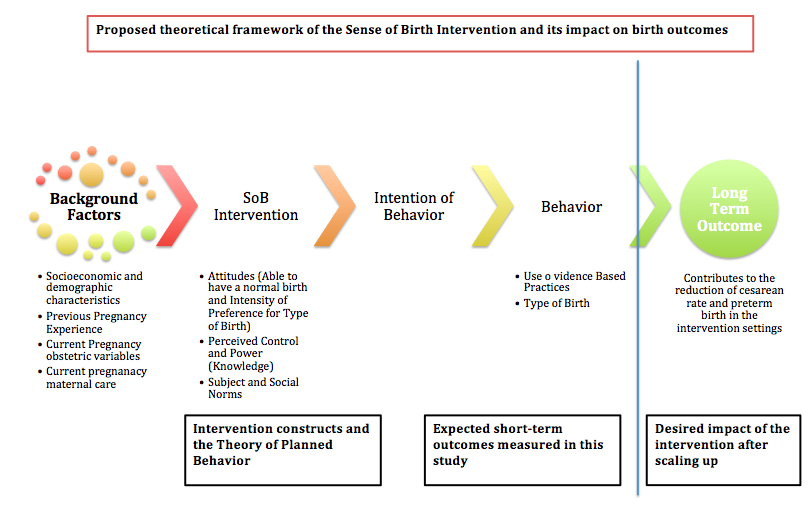
**
