# Supplementary material for: Brazilian women’s use of evidence-based practices in childbirth after participating in the Senses of Birth intervention: A mixed-methods study
Source: PLoS One. 2021 Apr 16;16(4):e0248740. doi: 10.1371/journal.pone.0248740 (PMC8051805; doi:10.1371/journal.pone.0248740)
Supplement: S2 File — (DOCX) [file pone.0248740.s002.docx]

# **S2 File – The Senses of Birth post-intervention and follow-up surveys to pregnant women – English version**

***Senses of Birth Pregnant Women Surveys***[1]

[1] L. da M. M. Fernandes, “Pregnant women’s knowledge and use of evidence-based practices during labor and childbirth after participating in a health education intervention – Senses of Birth,” State University of New York, 2019.

## ***Appendix 1 – Post-intervention survey for Pregnant Women*** ^[[1]](#footnote-1)^

**1. Date**

**2. Full name**

**3. What is your age?**

**4. What is your address?** Neighborhood / City / State

**5. What is your telephone number:** Landline / Cell phone / I do not want to provide

**6. What is your e-mail?**

**7. Marital Status**: Single / Married or civil union / Widow / Separated or divorced

**8. Family Income**^[[2]](#footnote-2)^

**(Refer to the sum of income of all the people who live in your household)**

1. Up to 1 minimum wage (<788.00)
2. From 1 to 2 minimum wages (2788.00 to 1576.00)
3. From 2 to 3 minimum wages (1576.00 to 2364.00)
4. From 3 to 5 minimum wages (2364.00 to 3,940.00)
5. From 5 to 10 minimum wages (3,940.00 to 7,880.00)
6. From 10 to 20 minimum wages (7,880.00 to 15,760.00)
7. More than 20 minimum wages (> 15,760.00)
8. I do not know / I do not want to inform

**9. Your work situation - check one option only**

1. Work with a formal contract
2. Works without a formal contract
3. Public servant (municipal, state, federal or military)
4. Employee – owns the business
5. Autonomous (includes MEI)
6. Cooperative
7. I have no work that earns money
8. Other (specify)

**10. What is your occupation? Check all options that apply**

1. Physician
2. Nurse
3. Other health professionals (please specify)
4. Teacher
5. Middle School Student
6. High School Student
7. Undergraduate Student
8. Graduate student
9. Housewife
10. Retired
11. Other (please specify)

**11. What is your schooling?**

1. Incomplete middle school
2. Complete middle school
3. Incomplete high school
4. Complete high school
5. Incomplete undergrad education
6. Completed undergraduate education and more
7. I do not want to inform

**12. The color of your skin is (read option)**

1. White
2. Black
3. Pardo/Mulato
4. Asian/Eastern
5. Indigenous

**13. Do you have private health insurance?** Yes / No

**14. How many times have you been pregnant before, excluding this pregnancy and counting termination (voluntary or natural)? You have had If answer = 0 move to question 17** - (Insert Number)

**15. How many births were normal (including birth using forceps and vacuum)?** (Insert Number)

**16. Moreover, how many were cesarean?** (Insert Number)

**17. How many weeks/months of gestation are you?**

Insert number for Weeks / Insert number for Months

**18. Is your pregnancy considered high risk?**

Yes / No

If yes, please describe why __________________

**19. Your prenatal appointments (current pregnancy) are covered by (check all that apply)**

1. Private Health Insurance
2. SUS (National Universal Public Health System)
3. Private (direct payment to professional – out of pocket payments)
4. I never had prenatal care
5. Other (please specify)

**20. Do you prefer NORMAL BIRTH?**

No way / a little / Maybe / Probably / Absolutely

**21. Do you prefer CESARIAN?**

No way / a little / Maybe / Probably / Absolutely

**22. Your knowledge about NORMAL BIRTH BEFORE the exhibition was:**

None / Poor / Fair / Good / Very good

**23. Your knowledge about a CESAREAN BEFORE the exhibition was:**

None / Poor / Fair / Good / Very good

**24. Your knowledge about NORMAL BIRTH AFTER the exhibition was:**

None / Poor / Fair / Good / Very good

**25. Your knowledge about a CESAREAN AFTER the exhibition was:**

None / Poor / Fair / Good / Very good

**26. BEFORE the exhibition, would you say your knowledge about DOULAS was:**

None / Poor / Fair / Good / Very good

**27. BEFORE the exhibition, would you say your knowledge about MIDWIVES/OBSTETRIC NURSES was:**

None / Poor / Fair / Good / Very good

**28. BEFORE the exhibition, would you say your knowledge about the Pregnant Woman's Right to have companionship, from her choice, during the labor and childbirth was:**

None / Poor / Fair / Good / Very good

**29. BEFORE the exhibition, would you say your knowledge about non-pharmacological birth pain relief methods was:**

None / Poor / Fair / Good / Very good

**30. Before the exhibition, would you say your knowledge about humanized and evidence-based care during labor and childbirth was:**

None / Poor / Fair / Good / Very good

**31. Before the exhibition, would you say your knowledge about organizations (NGOs, social networks, professionals, others) that defend the humanized and evidence-based care during labor and childbirth was:**

None / Poor / Fair / Good / Very good

**32. Before the exhibition, would you say your knowledge about the cesarean rates in Brazil was:**

None / Poor / Fair / Good / Very good

**33. Before the exhibition, would you say your knowledge about the Ministry of Health / World Health Organization guidelines for labor and childbirth care were:**

None / Poor / Fair / Good / Very good

**34. Before the exhibition, would you say your knowledge about Obstetric violence was:**

None / Poor / Fair / Good / Very good

**35. Before the exhibition, would you say your knowledge about Birth Plan was:**

None / Poor / Fair / Good / Very good

**36. Have you ever had experience with normal birth?**

1. Never had normal birth
2. Positive experience
3. Negative experience

Describe _________________________

**37. After the exhibition, would you say your knowledge about DOULAS is:**

None / Poor / Fair / Good / Very good

**38. After the exhibition, would you say your knowledge about MIDWIVES/OBSTETRIC NURSES is:**

None / Poor / Fair / Good / Very good

**39. After the exhibition, would you say your knowledge of the Pregnant Woman's Right to have companionship, from her choice, during the labor and childbirth is:**

None / Poor / Fair / Good / Very good

**40. After the exhibition, would you say your knowledge about non-pharmacological birth pain relief methods is:**

None / Poor / Fair / Good / Very good

**41. After the exhibition, would you say your knowledge about humanized and evidence-based care during labor and childbirth is:**

None / Poor / Fair / Good / Very good

**42. After the exhibition, would you say your knowledge about organizations (NGOs, social networks, professionals, others) that defend the humanized and evidence-based care during labor and childbirth is:**

None / Poor / Fair / Good / Very good

**43. After the exhibition, would you say your knowledge about the cesarean rates in Brazil is:**

None / Poor / Fair / Good / Very good

**44. After the exhibition, would you say your knowledge about the Ministry of Health / World Health Organization guidelines for labor and childbirth care are:**

None / Poor / Fair / Good / Very good

**45. After the exhibition, would you say your knowledge about Obstetric violence is:**

None / Poor / Fair / Good / Very good

**46. After the exhibition, would you say your knowledge about Birth Plan is:**

None / Poor / Fair / Good / Very good

**47. Your knowledge about the risks of NORMAL BIRTH BEFORE the exhibition was:**

None / Poor / Fair / Good / Very good

**48. Your knowledge about the risks of CESAREAN BEFORE the exhibition was:**

None / Poor / Fair / Good / Very good

**49. Your knowledge about the risks of NORMAL BIRTH AFTER the exhibition is:**

None / Poor / Fair / Good / Very good

**50. Your knowledge about the risks of CESAREAN AFTER the exhibition is:**

None / Poor / Fair / Good / Very good

**51. Did your preference for the type of birth (NORMAL BIRTH) change AFTER the exhibition?**

No way / a little / Maybe / Probably / Absolutely

**52. Did your preference for the type of birth (CESAREAN) change AFTER the exhibition?**

No way / a little / Maybe / Probably / Absolutely

**53. Do you think you can have a NORMAL childbirth?**

No way / a little / Maybe / Probably / Absolutely

**54. How will your labor and childbirth be paid? - Consider the various financing possibilities of the hospital and the professional who will attend the delivery (check all options that apply)**

1. Private Health Insurance
2. SUS (National Universal Public Health System)
3. Private (direct payment to professional – out of pocket payments)
4. Private Health Insurance + out of pocket only for the obstetrician
5. Other (please specify)

**55. If you had a previous cesarean section, identify the reason (s) - spontaneous response, check all options that apply.**

1. I did not have a previous cesarean
2. I wanted to connect the tubes
3. I wanted to have a cesarean
4. I had one cesarean before
5. I had two or more cesareans before.
6. I did not want to feel the pain of normal childbirth
7. I fear the of lack of beds for hospitalization
8. I fear the city violence
9. My baby was wrapped in the cord
10. My baby was crossed
11. My baby was sitting
12. My baby was too big / I had no passage / I had no dilatation (failure to progress)/ my baby did not settle into my pelvis
13. I passed my due date
14. My baby was growing too slow or stopped growing
15. My placenta was old
16. My baby was suffering
17. I did not have enough amniotic liquid
18. I had a low-lying placenta
19. I had high blood pressure
20. I had diabetes
21. I had problems with HIV / AIDS
22. I had a genital ulcer/condyloma or issue in the preventive uterus cervix exam
23. I had a positive result exam for *Streptococcus* in the vagina and/or anus
24. I had a premature placental abruption
25. I had a bleeding
26. My water broke
27. My labor never started
28. I was pregnant with twins
29. I had a fetal death
30. My pregnancy induce failed
31. I had a previous gynecologic surgery

Other (describe) ____________________________________________

**56. Did you have information about the benefits of normal birth during your prenatal care appointments?**

Yes / No / I did not have prenatal appointment

Specify____________________________________

**57. Do you participate in movement/e-mail lists/group discussions of pregnant women from humanized care to childbirth?**

Yes. Specify which ______________________________________

No

Other (specify)________________________________________

**58. AFTER the exhibition, have you changed your perception about the NORMAL BIRTH?**

No way / a little / Maybe / Probably / Absolutely

**59. After the exhibition, have you changed your perception of CESAREAN?**

No way / a little / Maybe / Probably / Absolutely

**60. BEFORE the exhibition you used to associate normal birth with JOY?**

Never / Rarely / Occasionally / Frequently / Always

**61. BEFORE the exhibition you used to associate normal birth with FEAR?**

Never / Rarely / Occasionally / Frequently / Always

**62. BEFORE the exhibition you used to associate normal birth with PAIN?**

Never / Rarely / Occasionally / Frequently / Always

**63. BEFORE the exhibition you used to associate normal birth with LOVE?**

Never / Rarely / Occasionally / Frequently / Always

**64. BEFORE the exhibition you used to associate normal birth with SUFFERING?**

Never / Rarely / Occasionally / Frequently / Always

**65. BEFORE the exhibition you used to associate normal birth with ANXIETY?**

Never / Rarely / Occasionally / Frequently / Always

**66. BEFORE the exhibition you used to associate normal birth with SAFETY?**

Never / Rarely / Occasionally / Frequently / Always

**67. BEFORE the exhibition you used to associate normal birth with CHALLENGE?**

Never / Rarely / Occasionally / Frequently / Always

**68. BEFORE the exhibition you used to associate normal birth with COURAGE?**

Never / Rarely / Occasionally / Frequently / Always

**69. BEFORE the exhibition you used to associate normal birth with RISK?**

Never / Rarely / Occasionally / Frequently / Always

**70. BEFORE the exhibition you used to associate normal birth with CONFIDENCE?**

Never / Rarely / Occasionally / Frequently / Always

**71. BEFORE the exhibition you used to associate normal birth with ACHIEVEMENT?**

Never / Rarely / Occasionally / Frequently / Always

**72. BEFORE the exhibition you used to associate normal birth with STRENGTH?**

Never / Rarely / Occasionally / Frequently / Always

**73. After the exhibition, you associate normal birth with JOY?**

Never / Rarely / Occasionally / Frequently / Always

**74. After the exhibition, you associate normal birth with FEAR?**

Never / Rarely / Occasionally / Frequently / Always

**75. After the exhibition, you associate normal birth with PAIN?**

Never / Rarely / Occasionally / Frequently / Always

**76. After the exhibition, you associate a normal birth with LOVE?**

Never / Rarely / Occasionally / Frequently / Always

**77. After the exhibition, you associate normal birth with SUFFERING?**

Never / Rarely / Occasionally / Frequently / Always

**78. After the exhibition, you associate normal birth with ANXIETY?**

Never / Rarely / Occasionally / Frequently / Always

**79. After the exhibition, you associate normal birth with SAFETY?**

Never / Rarely / Occasionally / Frequently / Always

**80. After the exhibition, you associate normal birth with CHALLENGE?**

Never / Rarely / Occasionally / Frequently / Always

**81. After the exhibition, you associate normal birth with COURAGE?**

Never / Rarely / Occasionally / Frequently / Always

**82. After the exhibition, you associate normal birth with RISK?**

Never / Rarely / Occasionally / Frequently / Always

**83. After the exhibition, you associate normal birth with CONFIDENCE?**

Never / Rarely / Occasionally / Frequently / Always

**84. After the exhibition, you associate normal birth with ACHIEVEMENT?**

Never / Rarely / Occasionally / Frequently / Always

**85. After the exhibition, you associate normal birth with STRENGTH?**

Never / Rarely / Occasionally / Frequently / Always

**86. What do you think influences your preference for the type of birth? Spontaneous response - Check all that apply**

1. Birth stories of your family and/or your friends
2. Your husband's preference for the type of birth
3. Fear of normal birth pain
4. Fear of normal birth change your vagina
5. I wanted to bind the tubes
6. Fear of cesarean section
7. Fear of anesthesia
8. To schedule the due date
9. Have a known professional at delivery
10. Positive previous experience with normal birth
11. Previous negative experience with normal birth
12. Positive previous experience with cesarean section
13. Previous negative experience with cesarean section
14. Online information
15. Information in newspaper and magazine
16. Information on television
17. Information on pregnant women groups
18. Normal childbirth is better than cesarean section
19. Better recovery in normal birth

Other (please specify) ______________________________

**87. How did you hear about the exhibit?**

1. Social networks (Facebook / Instagram / Twitter)
2. Newspaper / radio / television
3. Site
4. Friend/family
5. Posters / Brochures
6. Other (please specify):

__________________________________

**88. What brought you to the exhibition**

1. I was hanging around / waiting for someone/passing by
2. I came because it is free
3. I am interested in the subject
4. They recommended me
5. My institution (school/work/health center/others) has scheduled my visit

Other (please specify) _________

**89. Do you usually visit exhibitions and museums?**

Never / Rarely / On occasion / Frequently / Always

**90. In your opinion, the exhibition was:**

Bad / Regular / Good / Very good / Great

**91. What did you like best about the exhibition?**

1. Gestation (baby in the belly)
2. Surgical Maternity convenience store (product shelf)
3. Controversies (videos with dialogues / opinions)
4. Birth (birth tunnel)
5. Conversations (area of texts, photos, videos)
6. None of the option

**92. Do you intend to recommend this exhibition to others?**

No way / I think not / Perhaps / Most likely / Certainly.

## ***Appendix 2 – Follow-up survey***

**Pregnant Women Follow-Up Survey**^[[3]](#footnote-3)^

**SECTION I**

**1. Today's date: ________**

**2. Name /Last name ________**

**3. What was your date of birth? ________**

**4. How many weeks/months of gestation did you give birth? ________**

**5. Where did you birth take place?**

1. Private Hospital / Maternity
2. Public Hospital / Maternity - (SUS)
3. Birth House / Normal Birth Center
4. Residence
5. Other: ______________________

**5.1 Name of the hospital ________**

**6. Which was your type of birth?**

1. Normal/Vaginal
2. Cesarean section
3. Vaginal with use of forceps/vacuum extractor

**7. If cesarean, what was the reason? ________**

**7.1 If cesarean, when it occurred?**

1. Before Labor
2. During labor
3. I had a normal labor

**8. Have you had any memory of the Senses of Birth Exhibition during labor/delivery?** Yes / No

**8.1. Please comment: ________**

**9. Have the Senses of Birth Exhibition influenced your childbirth in any way? Rate how much: 1 (not at all) to 5 (much)**

1 / 2 / 3 / 4 /5

**9.1 Please comment ________**

**9.2 Was the influence positive?** Yes / No / Did not influence

**9.3 Please comment: ________**

**10. Evaluate your satisfaction with your delivery: Rate how much: 1 (very bad) to 5 (great)**

1 / 2 / 3 / 4 /5

**11. Do you consider that you have experienced violence/maltreatment during childbirth/cesarean section/birth of the baby?**

Yes / No / Do not know

**Please comment ________**

**12. Tell us a little about your birth experience: ________**

**SECTION II**

**1. Gestational age at birth was defined by**

1. Date of last menstruation
2. Ultrasound before 20 weeks
3. Estimated by obstetrician
4. Estimated by the pediatrician
5. Do not know
6. Other: _____________

**2. At the time of delivery, you were considered pregnant at risk?**

Yes / No / Do not know

**2.1. If yes, why ? ________**

**3. Did you make a Birth Plan during pregnancy? (Childbirth planning with your choices/desires)**

Yes / No / Did not know/do not know what it is

**3.1 If not, why? ________**

**3.2. If you had a Birth Plan, was there any part of it that happened as expected?**

1. Yes
2. No
3. I did not have a normal vaginal delivery
4. I did not give birth

**3.3 If you had a Birth Plan, do you consider that the clinical care received correspond to your desires?**

1. Yes
2. No
3. Partially
4. I did not give birth

**3.4 Please comment ________**

**4. During your birth/cesarean section, did you have a companion?**

1. Throughout the time/hospitalization
2. In labor
3. During anesthesia
4. At the time of delivery/cesarean section
5. In postpartum / cesarean section
6. I did not have a companion

**5. Have you used methods for pain relief in labor/delivery?**

1. Yes
2. No
3. I did not have labor

**5.1. What methods of pain relief did you use during labor?**

1. Ball
2. Massage
3. Shower
4. Bathtub
5. Analgesia/epidural (anesthesia)
6. Free movement throughout labor (stayed in the position you chose)
7. Movement during labor (walking / dancing / crouching / rebozo)
8. I have not used pain relief method
9. I did not have labor
10. Other: **________**

**5.2 Please comment ________**

**6. At the time of childbirth you were:**

1. Squatting
2. On the stool
3. Lying (gynecological position)
4. Recumbent (with footrest - semi-seated)
5. On hands and knees
6. Lying on your side
7. I had C-section
8. Other: ________________

**7. During labor / cesarean section did you have the assistance of which health professionals (check all that apply)**

1. Doula
2. Physician obstetrician
3. Obstetric nurse
4. Midwife
5. Pediatrician
6. I did not have health professional’s assistance
7. Other: ______________________

**8. Who attended your delivery / cesarean delivery was the same prenatal professional?**

1. Yes
2. No
3. I did not have professional assistance during childbirth

**9. At the time of delivery, did someone squeeze/climb onto your belly for the baby to be born (Kristeller maneuver)?**

1. Yes
2. No
3. Do not remember

**10. Was a vaginal cut made at the time of the baby being born? (Episiotomy)**

1. Yes
2. No
3. Do not know
4. Did not have a vaginal delivery

**11. Did you have an episiotomy?**

1. Yes
2. No
3. Do not know
4. I had no episiotomy

**12. Was your baby born well?**

1. Yes
2. No, he was stillborn
3. No, he had a problem.

**12.1 If the baby had any problems at birth, what happened? ________**

**13. If the baby was born dead, why? What happened? ________**

*If the baby was born dead, please go to the last question (23). If your baby was born alive, please continue to answer the following questions.*

**14. Have you and your baby had skin-to-skin contact immediately after birth? (Baby without clothes or fabrics on your body?)**

Yes / No

**14.1 If not, why? ________**

**14.2 Did you and your baby stay in skin-to-skin contact for the first hour after birth? (Baby without clothes or fabrics on your body)**

Yes / No

**14.3 If not, why? ________**

**15. Was the baby put to breastfeed the first hour after birth?**

Yes / No

**15.1 If not, why? ________**

**16. After birth did the baby stay with you all the time (did not go to another place as a nursery or ICU)?**

Yes / No

**16.1 If not, why? ________**

**17. After the birth was your baby hospitalized?**

Yes / No

**17.1 If yes, why? ________**

**17.2 If yes, how many days? ________**

**18. Is your baby well today?**

Yes / No

**18.1 If not, why?**

- Is hospitalized
- Is sick

Other: _______________

**18.2 Comment on what is happening/happened to the baby ________**

**19. Has the baby been breastfed?**

Yes / No

**19.1 If you breastfed, how long was it exclusive breast milk (without adding other milk or foods)? ________**

**19.2. Currently, the baby feeds on: (check all that apply)**

1. Breast milk
2. Other liquids (tea, juice)
3. Other milk (from cow, goat, soy, formula, other)
4. Fruits
5. Soup/solid food
6. Other:___________________

**19.3. If you are not breastfeeding, why? ________**

**20. Your baby uses or has used a pacifier**

1- Never used / 2- Has used / 3- Is using

**21. Your baby uses or used a baby bottle**

1- Never used / 2- Has used / 3- Is using

**22. Your baby uses or used a cup**

1- Never used / 2- Has used / 3- Is using

**23. Recalling the period of pregnancy, have you taken any measures to prevent ZIKA virus infection?**

No / Yes

**23.1 If not, why? ________**

**23.2 If yes, what measure (s)? ________**

1. Questions presented here were translated into English by the corresponding author, the original questionnaire was developed by the SOB research team and used in Portuguese [↑](#footnote-ref-1)
2. Income was measured using the monthly family earning measure by the country minimum wage (< 2 minimum wages (MW), 2 to < 5 MW, and 5 to < 10 MW, and ≥10 MW). One minimum wage at the time of the intervention was approximate U$224.14, and the federal government annually defines the value. [↑](#footnote-ref-2)
3. ***Questions presented here were translated into English by the author, the original questionnaire was developed by the research team and applied in Portuguese*** [↑](#footnote-ref-3)
