# Supplementary material for: Brazilian women’s use of evidence-based practices in childbirth after participating in the Senses of Birth intervention: A mixed-methods study
Source: PLoS One. 2021 Apr 16;16(4):e0248740. doi: 10.1371/journal.pone.0248740 (PMC8051805; doi:10.1371/journal.pone.0248740)
Supplement: S3 File — (DOCX) [file pone.0248740.s003.docx]

# **S3 File – The Senses of Birth post-intervention and follow-up surveys to pregnant women – Portuguese version**

*Sentidos do Nascer – Questionários das Mulheres Grávidas e Puérperas[1]*

[1] B. J. de Oliveira, S. Lansky, K. V. dos Santos, E. D. Pena, C. Karmaluk, and A. A. L. Friche, “Sentidos do Nascer: exposição interativa para a mudança de cultura sobre o parto e nascimento no Brasil,” *Interface - Comun. Saúde, Educ.*, vol. 24, 2020, doi: 10.1590/interface.190395.

***Anexo 1 – Questionário Grávidas – saída da exposição***

1. DATA

MM DD AAAA hh mm AM/PM

| - |  |
| --- | --- |

Date


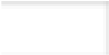


/


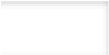


/


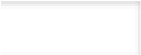

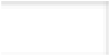


:


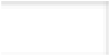


1. Nome completo


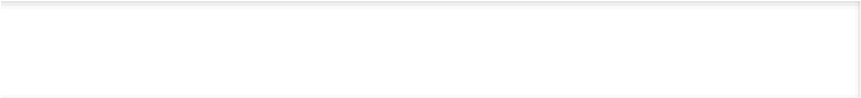


1. Qual é a sua idade?


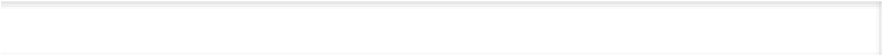


1. Qual é o seu endereço?

Bairro:


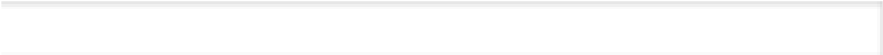

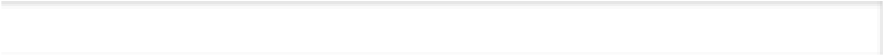


Município/Estado:

5. Qual é seutelefone

F

i

x

o

:


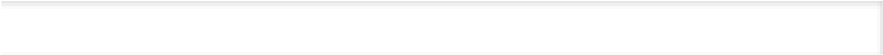


C

e

l

u

l

a

r

:


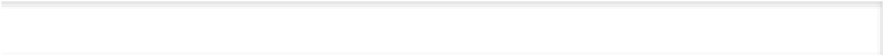


N

ã

o

q

u

e

r

o

f

o

r

n

e

c

e

r


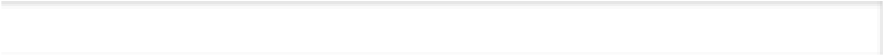


1. Qual o seu email?


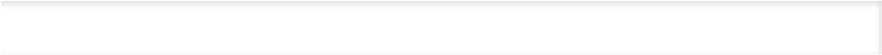


1. Estado Civil

Solteira


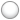

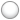

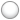

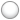


Casada ou união estável

Viúva

Separada ou divorciada

1. Renda familiar (Contando a renda de todas as pessoas que moram na sua casa)

Até 1 salário minimo (< 788,00)De 5 a 10 salários mínimos (3.940,00 a 7.880,00)


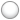

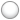

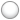

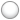

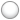

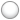

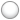

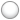


De 1 a 2 salários mínimos (788,00 a 1576,00)De 10 a 20 salários mínimos (7.880,00 a 15.760,00

De 2 a 3 salários mínimos (1576,00 a 2364,00)Mais de 20 salários mínimos (> 15.760,00)

De 3 a 5 salários mínimos (2364,00 a 3.940,00)Não sei/não quero informar

- 9. Sua situação de trabalho - assinalar uma opção apenas


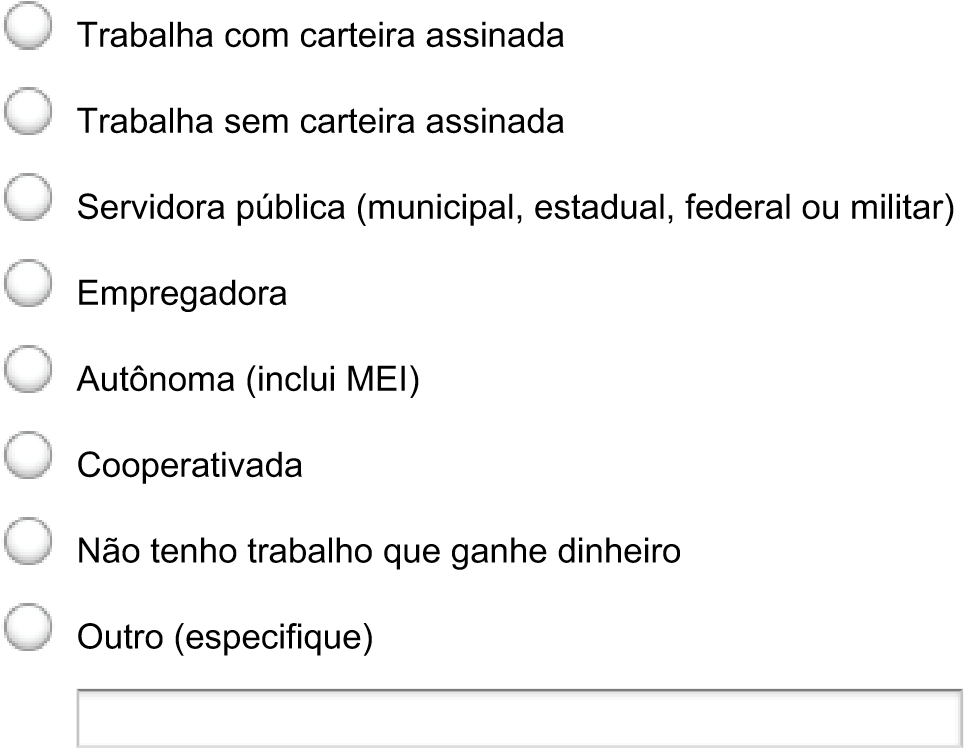


- 10. Qual a sua ocupação? Aceita mais de uma opção


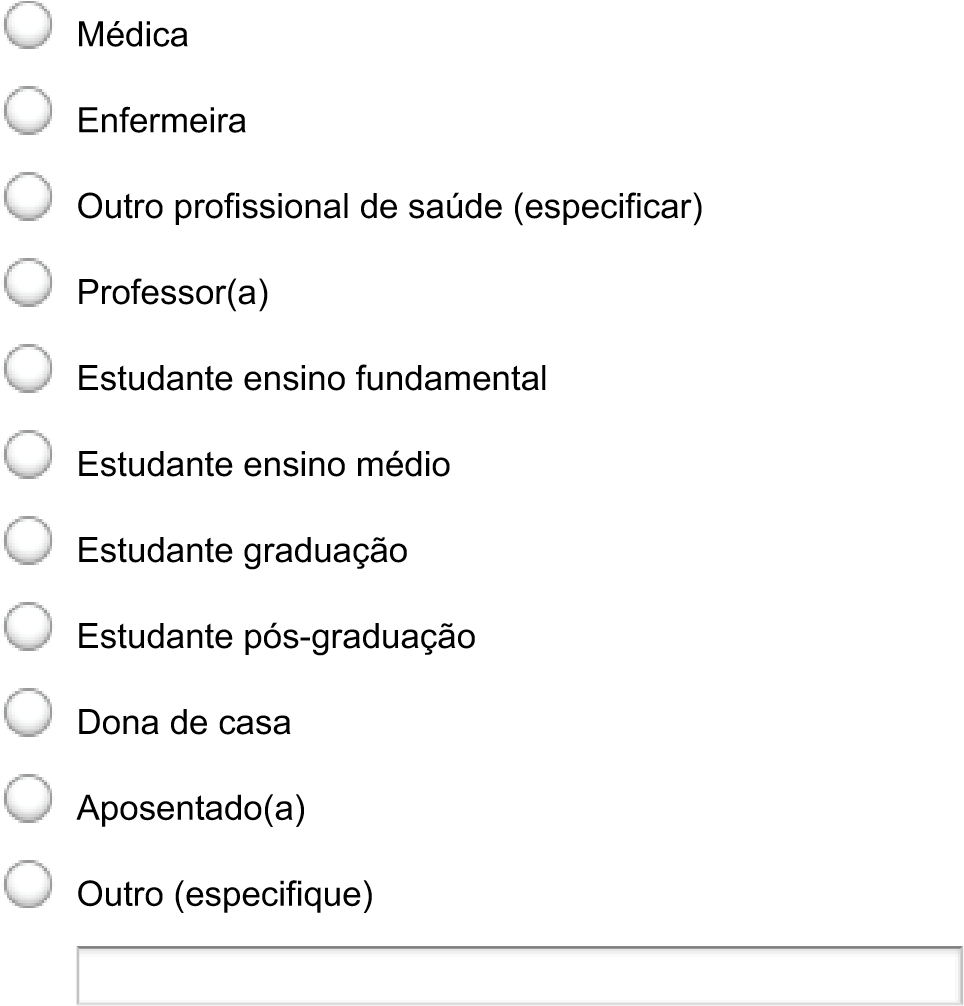


1. Qual a sua escolaridade

Ensino fundamental incompleto


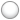

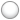

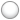

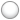

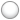

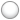

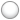


Ensino fundamental completo

Ensino médio incompleto

Ensino médio completo

Ensino superior incompleto

Ensino superior completo e mais

Não quero informar

1. A cor da sua pele é (ler as alternativas)

Branca


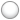

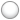

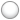

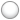

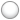


Preta

Parda/morena/mulata

Amarela (oriental)

Indígena

1. Você tem plano de saúde?

Sim


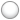

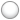


Não

* 14. Quantas vezes você ficou grávida excluindo esta gravidez e contando com algum aborto ou perda que você tenha tido? **Se resposta = 0 passar para pergunta 17**


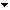

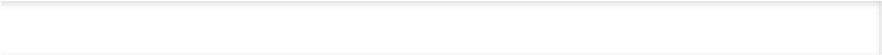


1. Quantos partos foram normais (incluindo partos a fórceps e vácuo)?


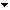

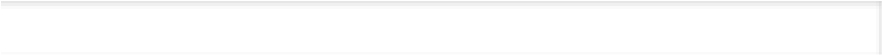


1. E quantos foram cesarianas?


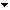

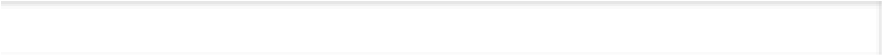


* 17. Com quantas semanas / meses de gestação você está?


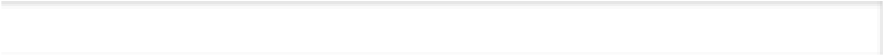

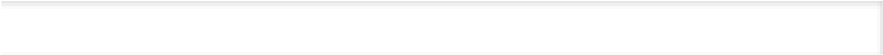


Semanas

meses

1. Você é considerada gestante de risco?

Sim


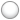

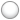

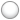


Não

Se sim, descreva porquê/sua condição______________________________________________________

1. O seu pré-natal (atual gravidez) é realizado em / no (aceita mais de uma opção)


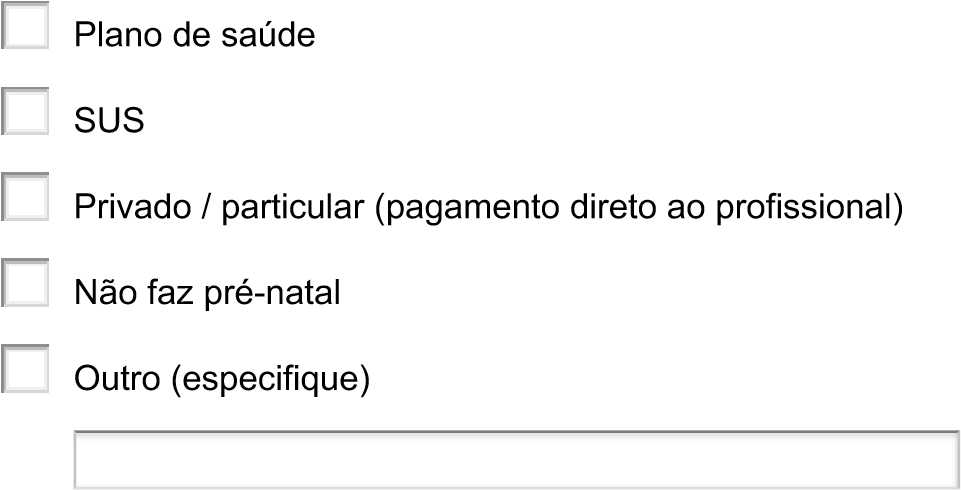


1. Você prefere ***PARTO NORMAL?***

De jeito nehum Um pouco Talvez Provavelmente Com certeza


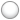

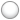

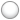

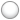

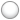


1. Você prefere ***CESARIANA?***

De jeito nehum Um pouco Talvez Provavelmente Com certeza


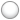

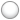

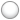

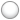

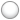


- 22. Seu conhecimento sobre o **PARTO NORMAL** ***ANTES* da exposição era:**

Nenhum Pouco Razoável Bom Muito bom


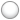

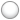

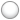

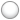

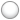


- 23. Seu conhecimento sobre a **CESARIANA *ANTES* da exposição era:**

Nenhum Pouco Razoável Bom Muito bom


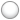

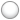

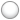

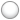

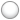


1. Seu conhecimento sobre o **PARTO NORMAL** ***APÓS*** a exposição é:

Nenhum Pouco Razoável Bom Muito bom


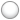

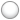

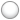

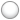

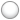


1. Seu conhecimento sobre a **CESARIANA** ***APÓS* a exposição é:**

Nenhum Pouco Razoável Bom Muito bom


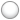

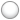

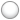

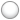

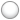


1. Antes da exposição você conhecia ***DOULA***:

Nem um pouco Pouco Razoavelmente Bem Muito bem


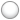

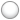

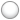

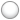

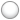


1. Antes da exposição você conhecia ***Enfermeira obstetra/obstetriz:***

Nem um pouco Pouco Razoavelmente Bem Muito bem


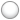

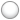

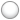

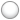

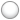


1. Antes da exposição você conhecia o ***Direito a acompanhante durante toda a internação para o parto:***

Nem um pouco Pouco Razoavelmente Bem Muito bem


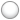

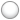

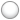

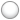

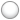


1. Antes da exposição você conhecia ***Outros métodos de alívio da dor do parto, além da anestesia***:

Nem um pouco Pouco Razoavelmente Bem Muito bem


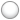

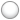


1. Antes da exposição você conhecia ***Assistência humanizada ao parto e nascimento***:

Nem um pouco Pouco Razoavelmente Bem Muito bem

1. Antes da exposição você conhecia ***Movimentos de humanização da assistência ao parto (ONG, grupos, redes sociais, profissionais, outros):***

Nem um pouco Pouco Razoavelmente Bem Muito bem

1. Antes da exposição você conhecia os ***Índices de cesariana no Brasil***:

Nem um pouco Pouco Razoavelmente Bem Muito bem

1. Antes da exposição você conhecia as ***Recomendações do Ministério da Saúde/ Organização Mundial da Saúde para a assistência ao parto:***

Nem um pouco Pouco Razoavelmente Bem Muito bem

1. Antes da exposição você conhecia ***Violência obstétrica / no parto:***

Nem um pouco Pouco Razoavelmente Bem Muito bem

1. Antes da exposição você conhecia ***Plano de parto***:

Nem um pouco Pouco Razoavelmente Bem Muito bem

1. Experiência com o parto normal prévio

N

ã

o

t

e

v

e

p

a

r

t

o

n

o

r

m

a

l

E

x

p

e

r

i

ê

n

c

i

a

p

o

s

t

i

v

a

E

x

p

e

r

i

ê

n

c

i

a

n

e

g

a

t

i

v

a

D

e

s

c

r

e

v

a

1. Depois da exposição você conhecia ***DOULA***:

Nem um pouco Pouco Razoavelmente Bem Muito bem

1. Depois da exposição você conhece ***Enfermeira obstetra/obstetriz:***

Nem um pouco Pouco Razoavelmente Bem Muito bem

1. Depois da exposição você conhece o ***Direito a acompanhante durante toda a internação para o parto:***

Nem um pouco Pouco Razoavelmente Bem Muito bem

1. Depois da exposição você conhece ***Outros métodos de alívio da dor do parto, além da anestesia***:

Nem um pouco Pouco Razoavelmente Bem Muito bem

1. Depois da exposição você conhece***Assistência humanizada ao parto e nascimento***:

Nem um pouco Pouco Razoavelmente Bem Muito bem

1. Depois da exposição você conhece ***Movimentos de humanização da assistência ao parto (ONG, grupos, redes sociais, profissionais, outros):***

Nem um pouco Pouco Razoavelmente Bem Muito bem

1. Depois da exposição você conhece os ***Índices de cesariana no Brasil***:

Nem um pouco Pouco Razoavelmente Bem Muito bem

1. Depois da exposição você conhece as ***Recomendações do Ministério da Saúde/ Organização Mundial da Saúde para a assistência ao parto:***

Nem um pouco Pouco Razoavelmente Bem Muito bem

1. Depois da exposição você conhece ***Violência obstétrica / no parto:***

Nem um pouco Pouco Razoavelmente Bem Muito bem

1. Depois da exposição você conhece ***Plano de parto***:

Nem um pouco Pouco Razoavelmente Bem Muito bem

1. Seu conhecimento sobre os riscos do **PARTO NORMAL *ANTES*** da exposição era

Nenhum Pouco Razoável Bom Muito bom

1. Seu conhecimento sobre os riscos da **CESARIANA *ANTES*** da exposição era

Nenhum Pouco Razoável Bom Muito bom

1. Seu conhecimento sobre os riscos do **PARTO NORMAL** ***APÓS*** a exposição é:

Nenhum Pouco Razoável Bom Muito bom

1. Seu conhecimento sobre os riscos da **CESARIANA** ***APÓS*** a exposição é:

Nenhum Pouco Razoável Bom Muito bom

1. Sua preferência sobre a via de parto (**PARTO NORMAL)** mudou ***APÓS*** a exposição?

De jeito nehum Um pouco Talvez Provavelmente Com certeza

1. Sua preferência sobre a via de parto (**CESARIANA)** mudou ***APÓS*** a exposição?

De jeito nehum Um pouco Talvez Provavelmente Com certeza

1. Você acha que conseguiria ter ***PARTO NORMAL***

De jeito nenhum Pouco provável Talvez Provavelmente Com certeza

1. Como será pago o seu parto - considerar as diversas possibilidades de financiamento do hospital e do profissional que assistirá o parto (aceita mais de uma opção)

P

l

a

n

o

d

e

s

a

ú

d

e

S

U

S

P

a

r

t

i

c

u

l

a

r

(

p

a

g

a

m

e

n

t

o

d

i

r

e

t

o

a

o

p

r

o

f

i

s

s

i

o

n

a

l

)

P

l

a

n

o

d

e

s

a

ú

d

e

+

p

a

g

a

m

e

n

t

o

p

a

r

t

i

c

u

l

a

r

(

d

i

r

e

t

o

a

o

p

r

o

f

i

s

s

i

o

n

a

l

)

O

u

t

r

o

(

e

s

p

e

c

i

f

i

q

u

e

)

1. Se teve cesariana prévia, identificar o(s) motivo(s) - resposta espontânea, aceita mais de uma opção

Não teve cesariana prévia Queria ligar as trompas

Queria fazer cesária

Já tinha cesárea uma anterior

Já tinha duas ou mais cesária

Não queria sentir a dor do parto normal

Medo de falta de vaga para internação

Medo da violência na cidade

Bebê estava enrolado no cordão

Bebê estava atravessado

Bebê estava sentado

Bebê era grande/ não tinha passagem/não tive dilatação/bebê não desceu/ não encaixou

Passou da hora / bebê passou do tempo

Bebê tava crescendo pouco ou parou de crescer

Sofrimento do bebê

Pouco líquido na bolsa (amniótico) Placenta baixa

Problema de pressão alta

Problema de diabetes

Infecção pelo HIV / AIDS positivo

Gravidez de gêmeos

Morte fetal

Verruga genital/condiloma ou problema no preventivo do colo do útero

Exame de cultura para streptococo na vagina e/ou ânus

Descolamento prematuro da placenta

Sangramento

Placenta velha

A bolsa rompeu

Não entrei em trabalho de parto

Falha de indução/ indução não funcionou

Cirurgia ginecológica anterior

Outra razão não citada (descrever)

____________________________________________

__

1. Você teve informação no pré-natal sobre os benefícios do parto normal?

E

s

p

e

c

i

f

i

q

u

e

S

i

m

N

ã

o

N

ã

o

f

i

z

c

o

n

s

u

l

t

a

d

e

p

r

é

-

n

a

t

a

l

1. Você participa de movimento/listas/rodas de gestantes de assistência humanizada ao parto ?

S

i

m

.

E

s

p

e

c

i

f

i

c

a

r

q

u

a

l

_

_

_

_

_

_

_

_

_

_

_

_

_

_

_

_

_

_

_

_

_

_

_

_

_

_

_

_

_

_

_

_

_

_

_

_

_

_

N

ã

o

O

u

t

r

o

(

e

s

p

e

c

i

f

i

q

u

e

)

1. ***APÓS*** a exposição, você mudou sua percepção sobre o ***PARTO NORMAL***

Nem um pouco Pouco Razoavelmente Muito Totalmente

1. ***APÓS*** a exposição, você mudou sua percepção sobre a ***CESARIANA***

Nem um pouco Pouco Razoavelmente Muito Totalmente

- 60. ***ANTES*** da exposição você associava o parto normal a ***ALEGRIA:***

Nunca Raramente De vez em quando Frequentemente Sempre

- 61.***ANTES*** da exposição você associava o parto normal a ***MEDO:***

Nunca Raramente De vez em quando Frequentemente Sempre

- 62.***ANTES*** da exposição você associava o parto normal a ***DOR:***

Nunca Raramente De vez em quando Frequentemente Sempre

- 63.***ANTES*** da exposição você associava o parto normal a ***AMOR:***

*64.***ANTES*** da exposição você associava o parto normal a ***SOFRIMENTO:***

Nunca Raramente De vez em quando

- 65.***ANTES*** da exposição você associava o parto normal a ***ANSIEDADE:***

Nunca Raramente De vez em quando Frequentemente Sempre

- 66.***ANTES*** da exposição você associava o parto normal a ***SEGURANÇA:***

Nunca Raramente De vez em quando Frequentemente Sempre

- 67.***ANTES*** da exposição você associava o parto normal a ***DESAFIO:***

Nunca Raramente De vez em quando Frequentemente Sempre

- 68.***ANTES*** da exposição você associava o parto normal a ***CORAGEM:***

Nunca Raramente De vez em quando Frequentemente Sempre

- 69.***ANTES*** da exposição você associava o parto normal a ***RISCO:***

Nunca Raramente De vez em quando Frequentemente Sempre

70.***ANTES*** da exposição você associava o parto normal a ***CONFIANÇA:***

Nunca Raramente De vez em quando

- 71.***ANTES*** da exposição você associava o parto normal a ***REALIZAÇÃO:***

Nunca Raramente De vez em quando Frequentemente Sempre

- 72.***ANTES*** da exposição você associava o parto normal a ***FORÇA:***

Nunca Raramente De vez em quando Frequentemente Sempre

- 73.***APÓS***  a exposição você associa o parto normal a ***ALEGRIA:***

Nunca Raramente De vez em quando Frequentemente Sempre

- 74.***APÓS*** a exposição você associa o parto normal a ***MEDO:***

Nunca Raramente De vez em quando Frequentemente Sempre

- 75.***APÓS*** a exposição você associa o parto normal a ***DOR:***

Nunca Raramente De vez em quando Frequentemente Sempre

76.***APÓS*** a exposição você associa o parto normal a ***AMOR:***

Nunca Raramente De vez em quando

- 77.***APÓS*** a exposição você associa o parto normal a ***SOFRIMENTO:***

Nunca Raramente De vez em quando Frequentemente Sempre

- 78.***APÓS*** a exposição você associa o parto normal a ***ANSIEDADE:***

Nunca Raramente De vez em quando Frequentemente Sempre

- 79.***APÓS*** a exposição você associa o parto normal a ***SEGURANÇA:***

Nunca Raramente De vez em quando Frequentemente Sempre

- 80.***APÓS*** a exposição você associa o parto normal a ***DESAFIO:***

Nunca Raramente De vez em quando Frequentemente Sempre

- 81.***APÓS*** a exposição você associa o parto normal a ***CORAGEM:***

Nunca Raramente De vez em quando Frequentemente Sempre

82.***APÓS*** a exposição você associa o parto normal a ***RISCO:***

Nunca Raramente De vez em quando

- 83.***APÓS*** a exposição você associa o parto normal a ***CONFIANÇA:***

Nunca Raramente De vez em quando Frequentemente Sempre

- 84.***APÓS*** a exposição você associa o parto normal a ***REALIZAÇÃO:***

Nunca Raramente De vez em quando Frequentemente Sempre

- 85.***APÓS*** a exposição você associa o parto normal a ***FORÇA:***

Nunca Raramente De vez em quando Frequentemente Sempre

1. O que você acha que influencia a sua preferência em relação ao tipo de parto?R esposta espontânea, aceita mais de uma

Experiência anterior negativa com parto normal

Experiência anterior positiva com cesariana

Experiência anterior negativa com cesariana

Informação na internet

Informação em jornal e revista

Informação na televisão

Informação em grupos de gestante

Parto normal é melhor que cesariana

Melhor recuperação no parto normal

1. Como você ficou sabendo da exposição?

Indicação de amigo(a) / família

R

e

d

e

s

s

o

c

i

a

i

s

(

F

a

c

e

b

o

o

k

/

i

n

s

t

a

g

r

a

m

/

t

w

i

t

t

e

r

)

J

o

r

n

a

l

/

r

á

d

i

o

/

t

e

l

e

v

i

s

ã

o

S

i

t

e

O

u

t

r

o

(

e

s

p

e

c

i

f

i

q

u

e

)

Vi quando passava por perto

Cartazes/folhetos

1. O que te trouxe à exposição?

1. Você costuma visitar exposições e museus?

Nunca

Raramente

De vez em quando

Com frequência

Sempre

1. Sua opinião sobre a exposição: Você achou a exposição:

Ruim Regular Boa Muito boa Ótima

1. O que você mais gostou da exposição?

A gestação (bebê na barriga)

Loja de conveniência da Maternidade Cirúrgica (prateleira dos produtos)

Controvérsias (vídeos com os diálogos / opiniões)

Nascimento (túnel do parto)

Conversas (área dos textos, fotos, vídeos)

Nenhuma das opções

1. Você pretende recomendar esta exposição para outras pessoas?

De jeito nenhum Acho que não Talvez Provavelmente Com certeza

***Anexo 2 - Sentidos do Nascer – Questionário pós-parto***

*Obrigatório

1. Data de hoje: *
2. Nome completo:*
3. Qual foi a data do seu parto? *
4. Seu parto ocorreu com quantas semanas/meses de gestação? *
5. Onde ocorreu o seu parto? * Hospital/Maternidade Particular Hospital/Maternidade SUS

Casa de Parto /Centro de Parto Normal (CPN) /Domicílio/ Outro:

- 1. **Nome do hospital ***

**6.Você teve parto** * Normal/Vaginal Cesárea**/** Vaginal com uso de fórceps/ Vácuo extrato

- 1. **Se cesariana, qual foi o motivo?**
  2. **Se cesariana, ocorreu** Antes do trabalho de parto/ Durante o trabalho de parto/ Tive parto normal

**7.Você teve alguma lembrança da Exposição Sentidos do Nascer durante o trabalho de parto / parto?** * Sim/ Não

7.1. Por favor, comente:

1. **A Exposição Sentidos do Nascer influenciou de alguma forma o seu parto? Avalie quanto: 1 (de jeito nenhum) a 5 (muito)** *

**8.1Por favor, comente**

- 1. **A influência foi positiva?** Não**/** Não influenciou
     1. **Por favor, comente:**

1. **Avalie a sua satisfação com o seu parto: 1 (péssimo) a 5 (ótimo)** *
2. **Você considera ter vivido violência/maus tratos no parto/cesariana/no nascimento do bebê?** * Sim/ Não/ Não sei

10.1Por favor, comente

1. **Conte-nos um pouco sobre sua experiência de parto:** *

PARTE II

1. **A idade gestacional no parto foi definida por / pela:**

1. Data da última menstruação
2. Ultrasom antes de 20 semanas
3. Estimada pelo obstetra
4. Estimada pelo pediatra
5. Não sei
6. Outro
7. **No momento do parto, você foi considerada gestante de risco?** Sim/Não/ Não sabe
   1. **Se sim, por quê?**
8. **Você fez plano de parto durante a gravidez? (planejamento do parto com as suas escolhas /desejos)** Sim/ Não fiz/ Não conhecia/ Não sei o que é

**3.1 Se não, porquê?**

3.2. Se você fez plano de parto, alguma parte dele foi realizada? Sim/ Não/ Não tive parto normal/vaginal/ Não fiz plano de parto

- 1. **Se você fez plano de parto, você considera que a assistência recebida correspondeu ao seu plano de parto?** Sim / Não**/** Parcialmente**/** Não fiz plano de parto
  2. **Por favor, comente**

**4.Durante o seu parto / cesariana você teve acompanhante?**

1. Durante todo o tempo / internação
2. No trabalho de parto
3. Durante a anestesia
4. Na hora do parto /cesariana
5. No pós-parto / cesariana
6. Não tive acompanhante

**5.Você usou métodos para alívio da dor no trabalho de parto/parto?**

Sim/Não/ Não tive trabalho de parto

**5.1Quais métodos de alívio da dor você utilizou durante o trabalho de parto?**

1. Bola
2. Massagem
3. Chuveiro
4. Banheira
5. Analgesia / peridural (anestesia)
6. Livre movimentação durante o trabalho de parto (ficou na posição que escolheu)
7. Movimentação durante o trabalho de parto (andar/dançar/agachar/rebozo)
8. Não usei método de alívio da dor
9. Não tive trabalho de parto
10. Outro:
    1. Por favor, comente
11. **Na hora do parto você ficou:**
12. No banquinho
13. Deitada (posição ginecológica)
14. Recostada (com apoio dos pés - semi-sentada)
15. De quatro
16. De lado
17. Tive cesariana
18. Outro
19. **Durante o trabalho de parto/parto/cesariana você teve assistência/acompanhamento de:**
20. Doula
21. Médico obstetra
22. Enfermeira obstetra
23. Obstetriz (parteira formada)
24. Pediatra
25. Não tive assistência de profissional
26. Outro:

**8.Quem atendeu seu parto/cesariana foi o mesmo profissional do pré-natal?** Sim/ Não/ Não tive assitência profissional no parto

**9.Na hora do parto, alguém apertou/subiu na sua barriga para a saída do bebê (manobra de Kristeller)?** * Sim/ Não/ Não se lembra

**10. Foi realizado um corte na vagina na hora do bebê nascer?** (episiotomia) Sim/ Não/ Não sabe/ Não teve parto vaginal

**10.1 Se teve o corte (episiotomia), você foi informada que esse corte seria feito?** Sim/ Não/ Não sabe/ Não teve episiotomia

**11.Seu bebê nasceu bem?** * Sim/ Não, ele foi natimorto (nasceu sem vida)/ Não, teve problema

11.1 Se o bebê teve algum problema ao nascer, o que aconteceu?

11.2 Se nasceu morto, qual o motivo, o que aconteceu?

11.2.1Se o bebê nasceu morto, por favor vá para a última questão (22). Se nasceu vivo, por favor continue respondendo as próximas questões.

**12. Você e seu bebê tiveram contato pele a pele imediatamente após o nascimento? (Bebê sem roupas ou panos no seu corpo?)** Sim/Não

**12.1Se não, por quê?**

**13.O bebê foi colocado para mamar na primeira hora após o nascimento?** Sim/ Não

**13.1 Se não, por quê?**

**14.Após o nascimento o bebê ficou todo o tempo junto com você?** Sim/Não

14.1 Se não, por quê?

**15.Após o nascimento seu bebê ficou internado?** Sim/ Não

**15.1Se sim, por quê?**

**15.2Se sim, quantos dias?**

**16.Hoje seu bebê está bem?** Sim/ Não

**16.1Se não, porque?** Está internado/ Está doente/ Outro:

- - 1. **Comente o que está acontecendo / aconteceu com o bebê**

**17.O bebê foi amamentado?** Sim/ Não

17.1 Se amamentou, quanto tempo de leite materno exclusivo (sem adição de outros leites ou alimentos)?

17.2 Se não está amamentando, por quê?

**18.** **Atualmente o bebê se alimenta com**

1. Leite materno
2. Outros líquidos (chá, suco)
3. Outros leites (de vaca, de cabra, de soja, fórmula,outro)
4. Frutas
5. Sopinha / comida de sal
6. Outro:

**19.Seu bebê usa ou usou Chupeta?**

1. Nunca usou
2. Já usou
3. Está usando

**20.Seu bebê usa ou usou Mamadeira?**

1. Nunca usou
2. Já usou
3. Está usando

**21.Seu bebê usa ou usou copo?**

1. Nunca usou
2. Já usou
3. Está usando

**22.Relembrando o período da gravidez,você tomou alguma medida de prevenção da infecção pelo vírus ZIKA?** * Não/Sim

**22.1Se não, por quê? ***

**22.2Se sim, qual (is) medida(s)?**
