# Supplementary material for: Brazilian women’s use of evidence-based practices in childbirth after participating in the Senses of Birth intervention: A mixed-methods study
Source: PLoS One. 2021 Apr 16;16(4):e0248740. doi: 10.1371/journal.pone.0248740 (PMC8051805; doi:10.1371/journal.pone.0248740)
Supplement: S4 File — (DOCX) [file pone.0248740.s004.docx]

# **S4 File – Quantitative Measures used in the “Brazilian women’s use of evidence-based practices in childbirth after participating in the Senses of Birth intervention: a mixed-methods study.”**

Detailed description of the quantitative variables are a support information of the above referred article and part Thesis “Pregnant women knowledge and use of evidence-based practices during labor and childbirth after participating in a health education intervention – Senses of Birth”[1]

[1] L. da M. M. Fernandes, “Pregnant women’s knowledge and use of evidence-based practices during labor and childbirth after participating in a health education intervention – Senses of Birth,” State University of New York, 2019.

*Quantitative Measures used:*

1) **Socio-demographic characteristics:** Age (19 to 34 years old and ≥ 35 years old), race (white and black [Pardo/black] and other [Asian and indigenous]), education (< 12 years, 12 years), private health insurance (yes, no), and income (< 2 minimum wages (MW), 2 to < 5 MW, and 5 to < 10 MW, and ≥ 10 MW). Income was measured using the monthly family earnings relative to the country minimum wage. One minimum wage at the time of the intervention was approximately U$224.14.

2) **Obstetric characteristics**: First pregnancy (yes, no), type of hospital (SUS {public health system}, and Private [Private Health Insurance/Out of Pocket]), type of birth (vaginal birth, and cesarean), perceived ability to have a normal birth (yes, no).

The variable “type of hospital” classified the maternity hospitals where women gave birth based on the source of funding, a specific classification relevant to Brazil’s health system organization. “SUS” refers to public or non-profit hospitals funded by the public health system. "Private" refers to private or non-profit hospitals paid for by privately owned health insurance or out of pocket by the patients. Mixed funded hospitals were included in the public health system for this analysis.

3) **Perceived Knowledge.** Participants were asked to self-report their knowledge, before and after the intervention, with regard to: normal birth; cesarean; risks of normal birth; risks of cesarean; doula support; midwife care; companionship of her choice throughout the hospital stay, during labor and childbirth; access to non-pharmacological birth pain relief methods; birth plan; childbirth best practices; organizations that defend the humanized and evidence-based care model; Brazil's C-section rate; Ministry of Health (MS) and WHO guidelines for labor and childbirth care; and obstetric violence. Response options were chosen from a Likert scale, with the possible answers ranging from none (1) to very good (5).

The perceived knowledge variables were grouped into three different domains, based on the results of factor analysis of a previous study [41]. The domains are: 1) EBP Knowledge, 2) Normal Birth Knowledge, and 3) Cesarean Knowledge. For each knowledge domain, a mean score before the intervention and a mean score after the intervention were computed from the sum of all variables in that domain, ranging from 1 to 5 points [41]. A specific change score was calculated for each domain, representing the women’s perceived variation of knowledge using the difference between the mean after and the mean before, ranging from -5 to 5 [41]. Women who did not perceive an increase in knowledge after the intervention, presenting a score within -5 and 0, included those who had lower knowledge before the SoB intervention and whose knowledge did not increase, and women who ranked high on the scale of perceived knowledge before the intervention and did not perceive changes after it. Women who perceived knowledge increase after the intervention ranked between 0.1 and 5 [41].

**4) Use of Intrapartum EBP**. Participants were asked whether or not they used each of the evidence-based practices during labor and delivery: Birth Plan (yes, no [no, don’t know]); companionship during childbirth (yes, no [no, partially]); doula support (yes, no); midwife care (yes, no); freedom of mobility during labor (yes, no); choice of position during delivery (yes, no); use of non-pharmacological methods for pain relief (yes [exclusively and the use combined with pharmacological methods], no [no pain relief methods used or used only pharmacological methods]).

The EBP for continuum support is observed here with two variables: doula support and companionship during childbirth. Non-pharmacological methods of pain relief used by the women in this study were massage, birth ball, shower, bathtub, electrodes (TENS), music, meditation, and breathing techniques. Freedom of mobility during labor was characterized as walking, dancing, and crouching. Choice of position during delivery was characterized as any choice other than supine (traditional gynecological position), described by the woman as using a stool, use of bars, kneeling, semi-sitting with support, and sitting upright.
