# Supplementary material for: Brazilian women’s use of evidence-based practices in childbirth after participating in the Senses of Birth intervention: A mixed-methods study
Source: PLoS One. 2021 Apr 16;16(4):e0248740. doi: 10.1371/journal.pone.0248740 (PMC8051805; doi:10.1371/journal.pone.0248740)
Supplement: Attachment — Submitted filename: 0-COMMENTARY.docx [file pone.0248740.s005.docx]

**COMMENTARY**

From the methodological point of view, it is a study with a mixed design of quanti-quali character. The sample is considered appropriate for both methods and the statistical analysis is adequate. PAHO considers that the average number of Caesarean sections acceptable should be 15%. However, we know that there are absolute and relative indications for a Cesarean section. Within these conditions, we know that some social determinants are important and could explain some percentage of these cesarean sections, such as poverty, access to food and poor prenatal care, among other factors. In light of these factors, it should be considered whether this indicator of 15% should be raised and not force a reduction in the number of cesarean sections. This implies emphasizing the social determinants and those direct causes that could influence the decision of the mother or the doctor to have a Cesarean section instead of a natural birth. Pregnant women should be intervened from their first birth control, trying to guide them towards a natural birth.

On the other hand, it would always be important to have the instruments that were used to carry out the studies and in this way be able to know the variables and other factors that were considered and not only those that had positive results.

Dr. Jairo Vanegas López

Facultad Ciencias Médicas

Universidad de Santiago Chile
