# Supplementary material for: Brazilian women’s use of evidence-based practices in childbirth after participating in the Senses of Birth intervention: A mixed-methods study
Source: PLoS One. 2021 Apr 16;16(4):e0248740. doi: 10.1371/journal.pone.0248740 (PMC8051805; doi:10.1371/journal.pone.0248740)
Supplement: Attachment — Submitted filename: renamed_4d557.docx [file pone.0248740.s006.docx]

Santiago, February 11, 2020

Editors

Plos One

The manuscript has considered the requirements as established by PLOS ONE. The methodological issues were overcome by expanding the requested explanations and the limitations observed in the study were added. This will help to have a better understanding of the scope of the study itself. The sample issue was clarified, and some terms and table numbering were corrected. The issues related to the ethics committee were clarified.

Dr. Jairo Vanegas

Universidad de Santiago de Chile
